# Supplementary material for: The New GPI-Anchored Protein, SwgA, Is Involved in Nitrogen Metabolism in the Pathogenic Filamentous Fungus Aspergillus fumigatus
Source: J Fungi (Basel). 2023 Feb 15;9(2):256. doi: 10.3390/jof9020256 (PMC9960506; doi:10.3390/jof9020256)
Supplement: Supplementary file 1 [file jof-09-00256-s001.zip › jof-2172223-supplementary.pdf]

Figure S1

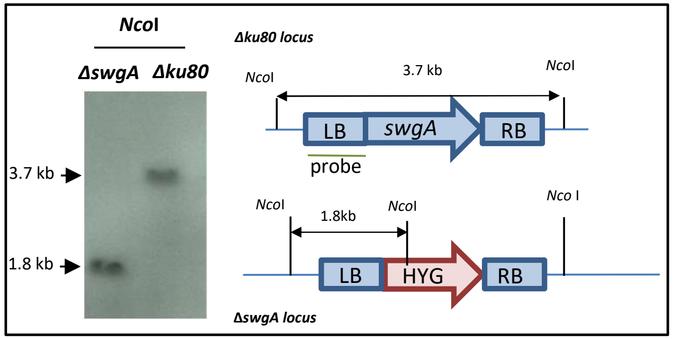

NcoI
ΔswgA Δku80
3.7 kb
1.8 kb
Δku80 locus
NcoI
3.7 kb
NcoI
LB
swgA
RB
probe
NcoI
1.8kb
NcoI
Nco I
LB
HYG
RB
ΔswgA locus

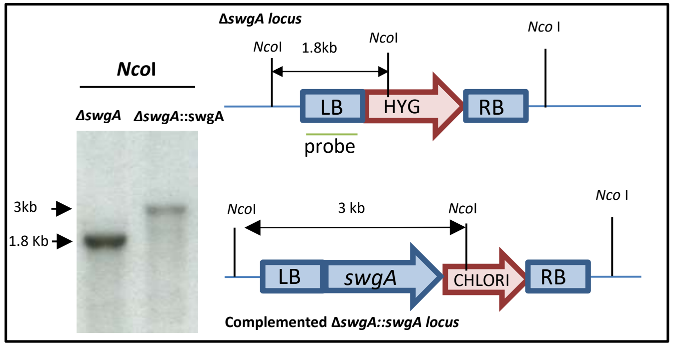

ΔswgA locus
NcoI
1.8kb
NcoI
Nco I
NcoI
ΔswgA ΔswgA::swgA
LB
HYG
RB
probe
3kb
1.8 Kb
NcoI
3 kb
NcoI
Nco I
LB
swgA
CHLORI
RB
Complemented ΔswgA::swgA locus

Figure S2

```
                    *        20         *        40         *       60         *       80
A.fumigatu : MVRFSLLLLASAISLTATASRLKRDTLKPFQEGRFAPSNNASTSLAYDNNLLPRYYDCLPGAHLCDAGQCCDFTCCSDGS :  80
A.fisheri  : MARFSFLLLASTISLTTTASSLKRDTLKPFQNGRFAPSNNASTSLAYDNNLLPRYYDCLPGAHLCDAGQCCDFTCCSDGS :  80
A.clavatus : MARISFLALASVIILNTSATSLERDTLKPFLTARFSPSGNESVSQAFENDLVPRYLSCTGGAHLCRAGQCCLYSCCSDGS :  80

                    *       100         *       120         *       140         *       160
A.fumigatu : CCGAAELCYNDVKPSKGPTYCCKIYTEKECDTRCVPMMSECCGDGYYCKWGHTCSGSYDDDDD---VGSGGDSGDYDSTT : 157
A.fisheri  : CCPAAQLCYNDVKPSKGPTYCCEIYTEKECDTRCVPMMSECCGNGFYCKWGHTCSGSYDDDDDDDDVGSGGGSKDHDSTT : 160
A.clavatus : CCGAGELCYNDKKPSKGPTYCCAMYTEKQCDTGCVPMQSECCGNGYYCRWGRTCRGGYGGTDSDDDDDDHETTTERTTRT : 160

                    *       180         *       200         *       220         *       240
A.fumigatu : SSTSYDLTTSYTSTEREVSTTSEYTTRTEERTTTSESSTRTRATDTDDAWETTETDSSTSTSTSSRWTMTDFPTGLDFTP : 237
A.fisheri  : S----------MSTERKVSTTSEYTTHTKERTTTSERSTRTRSTDTDGASETTETDSSTSTSTSSRRTTTDFPTASDFTP : 230
A.clavatus : YS---------PETESGSSSTSTSTSTSRESYTITETKSGRRSTSTDSSEITTSSSSSSDASESTS--SSRTTSTTRFTG : 229

                    *       260         *       280
A.fumigatu : AQSFVPAPVPTGPVSLSGANKNSLSSLDMGLLVIVTGAGWYLAL : 281
A.fisheri  : ARSFVPAPAPTGTVSLSGANKNSLSSLDMGLLVIVTGTGWYFAL : 274
A.clavatus : VATALAAPNPHSQAGDSAASSHSTS-LNTRFIVILAGILGYLRL : 272
```

Figure S3

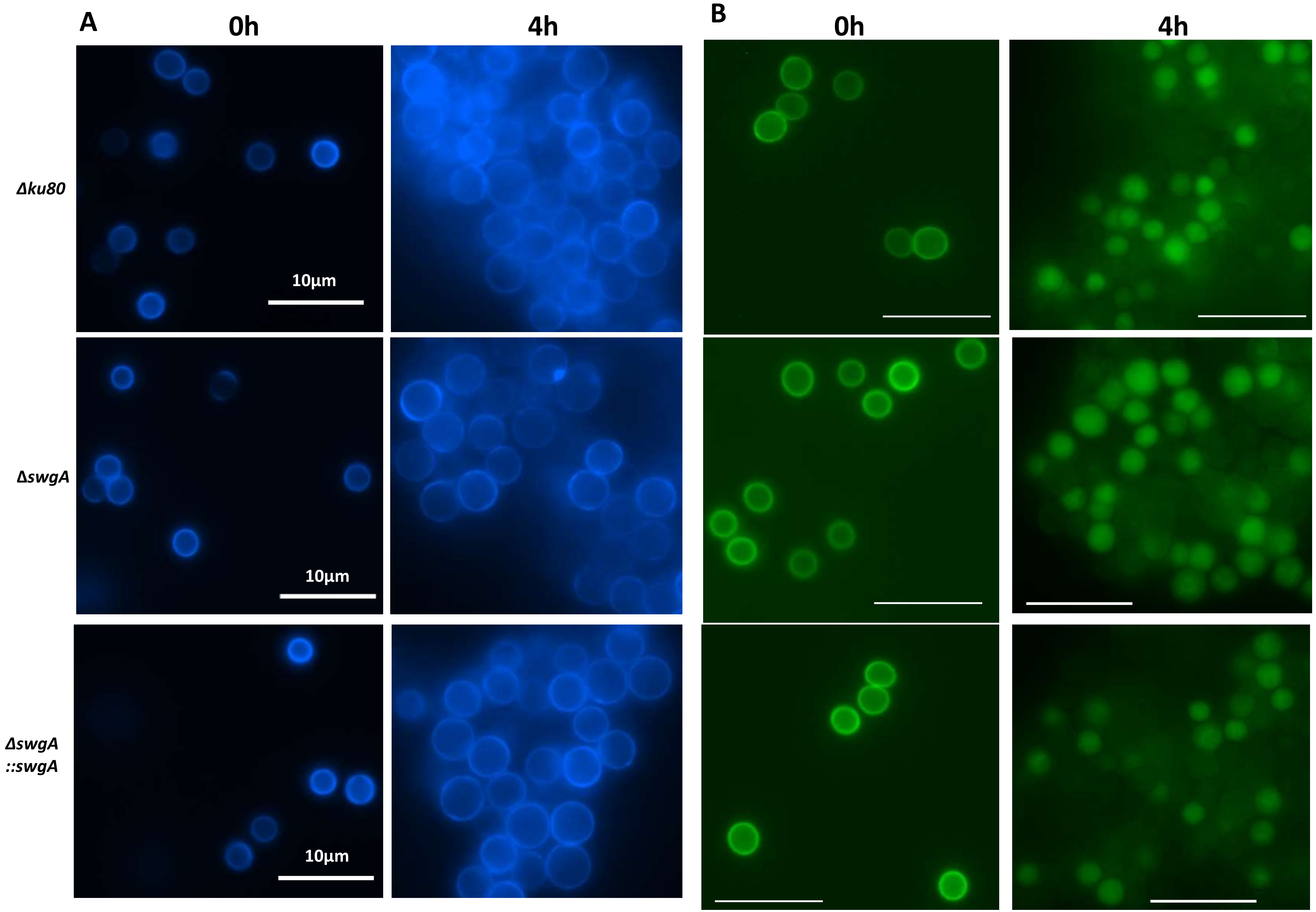

Figure S4

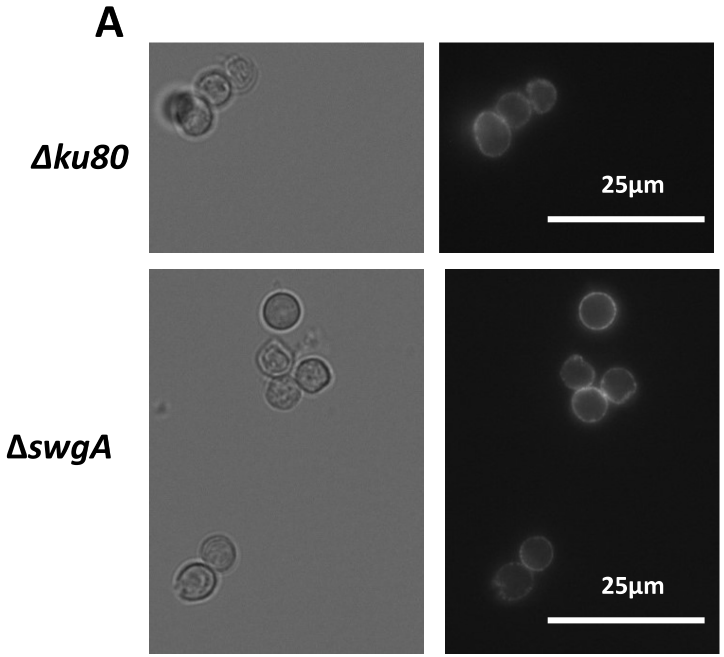

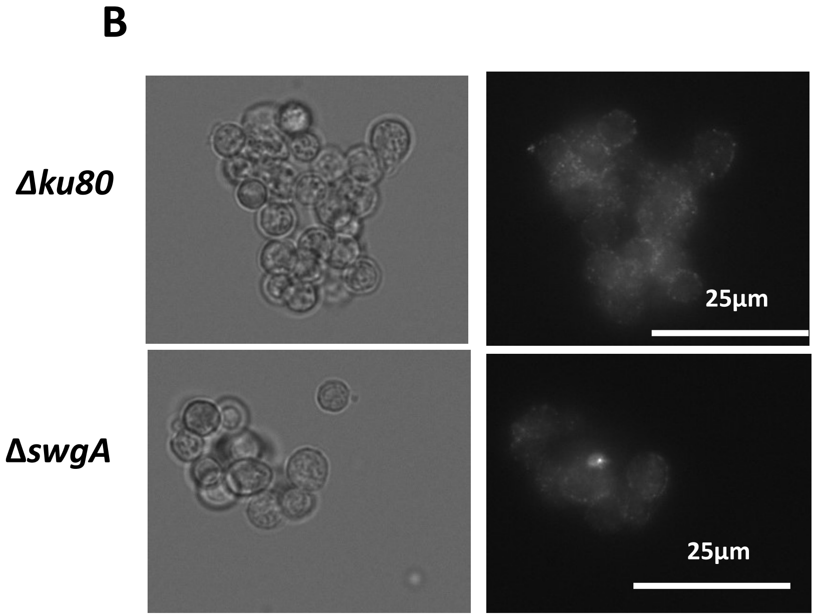

Figure S5

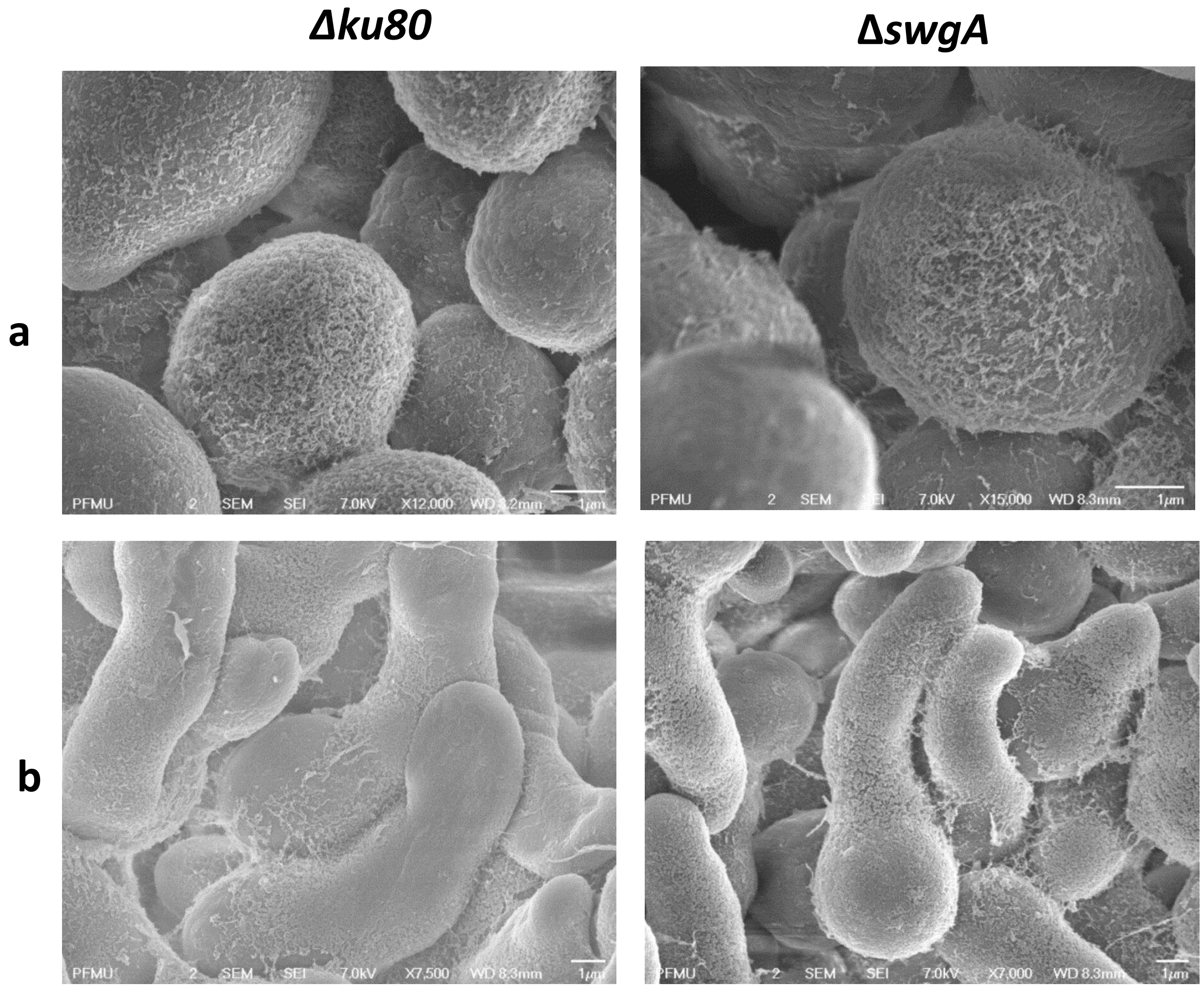

Figure S6

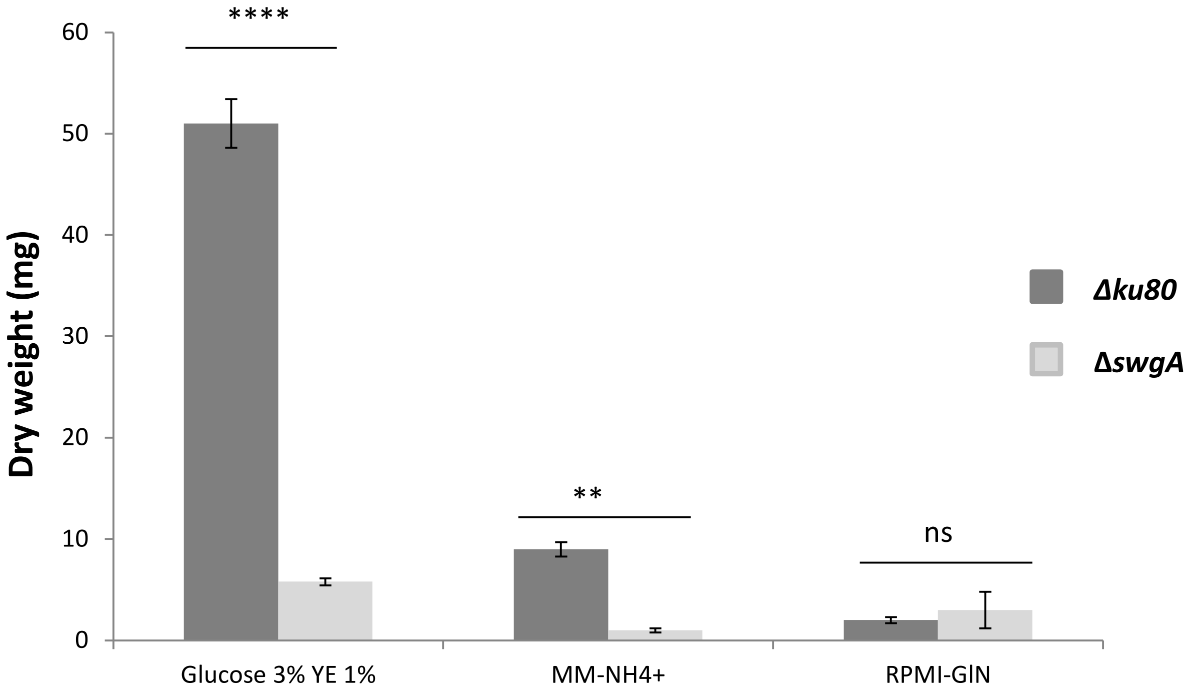

Dry weight (mg)
60
50
40
30
20
10
0
****
**
ns
Δku80
ΔswgA
Glucose 3% YE 1%
MM-NH4+
RPMI-GlN

Figure S7

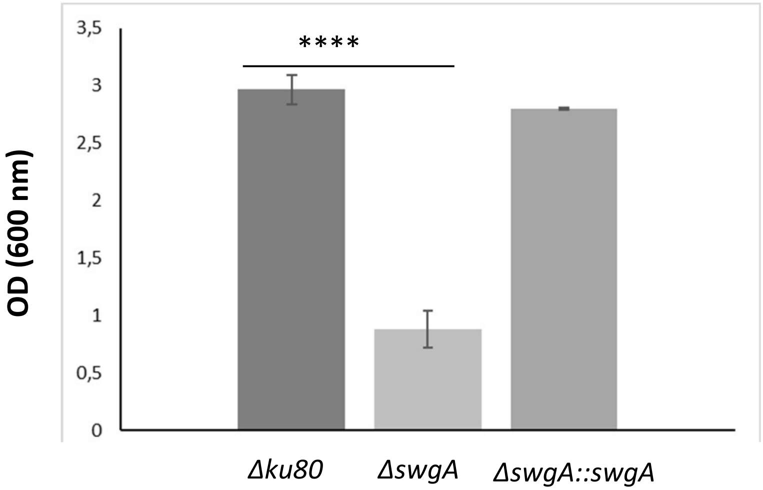

Figure S8

A

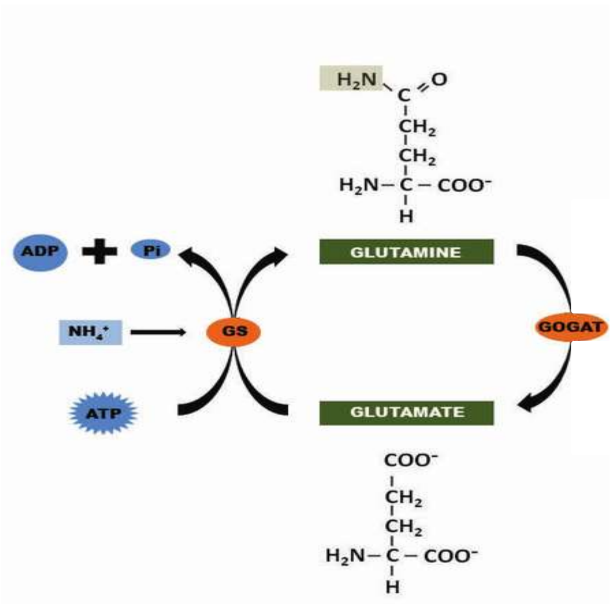

GS: Glutamine synthase ;
GOGAT:Glutamine Oxoglutarate Aminotransferase

B

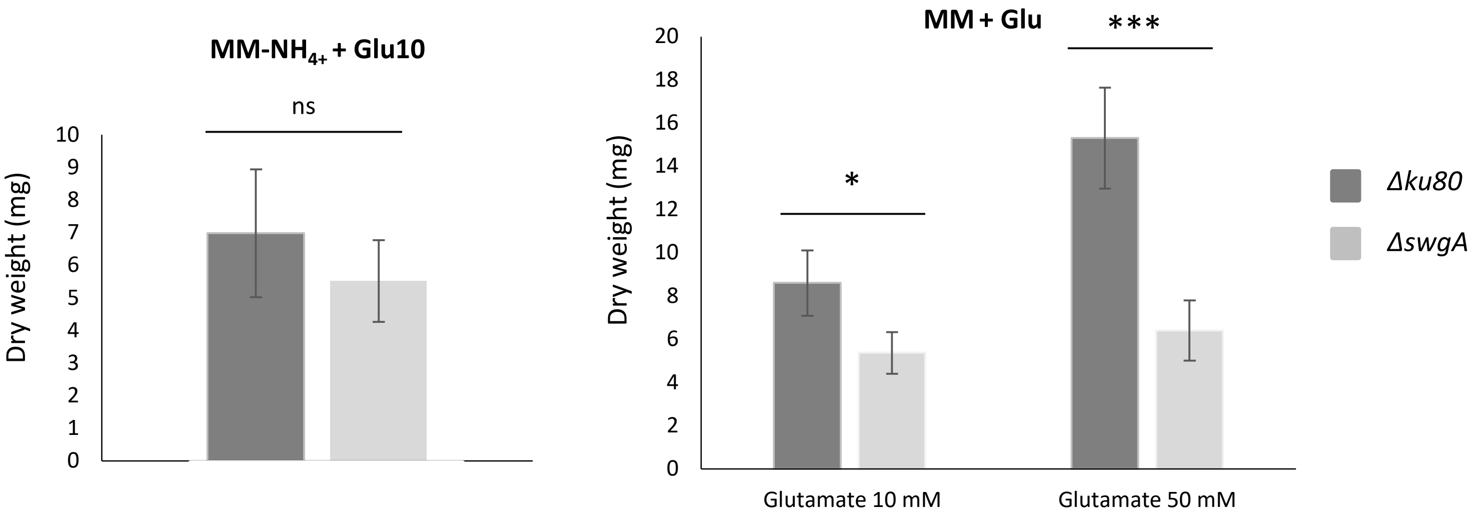

Table S1 : Primers used in this study

Name	Sequence	
*swgA*compF1	AATTCGAGCTCGGTACT**GCGCAG**ACACACGCCTGTTGGGTAT	
*swgA*compR1	GGACCTGAGTGATGCACCGATCACAATGCGAGATA	
*swgA*compF2	TGGTCCATCTAGTGCTCGGTGGACGACCAGGTAGATCACGAGCCACCTGC	
*swgA*compR2	GCCAAGCTTGCATGCCTGCGCACATGGTGTCTACCCCTTGCT	
areA-RTF	CGACCGACTCCAACCGCTGT	
areA-RTR	GGCGGGCGACTCGATAGGAA	
swgA-RTF	CTCCACCTCGCTAGCATACG	
swgA-RTR	TAGACACTTCCCGCTCCGTA	

Table S2: Characteristics of the protein encoded by the AFUA_8G01170 gene and orthologs in others species

**Species**	**Size (amino-acids)**	**Signal Peptide**	**GPI-anchor**	**Gene accession number**
A.fumigatus AF293	281	18: TAT-AS	254 SGA	AFUA_8G01170
A.fumigatus A1163	281	19: ATA-SR	254 SGA	AFUB_084830
A.fischeri	274	18: TTT-AS	246 SGA	XP_001261813.1
A.clavatus	272	19: TSA-TS	245 DSA	XP_001275113.1

Table S3: Percentage of identities between the different proteins: XP_001275113.1-*A.clavatus*; AFUA_8G01170-*A.fumigatus*; XP_001261813.1-*A.fisheri.*

	A.fumigatus	*A.fisheri*
A.clavatus	43	44
A.fumigatus		**84**
